# Bilirubin as a Protective Factor for Rheumatoid Arthritis: An NHANES Study of 2003 - 2006 Data

**DOI:** 10.4021/jocmr444w

**Published:** 2010-12-11

**Authors:** Daniel Fischman, Ashok Valluri, Venkata Subhash Gorrepati, Megan E. Murphy, Ian Peters, Pramil Cheriyath

**Affiliations:** a Pinnacle Health/Harrisburg Hospital, 205 S Front Street, Harrisburg, PA 17104, USA; b Philadelphia College of Osteopathic Medicine, Philadelphia, PA, USA

## Abstract

**Background:**

Rheumatoid arthritis(RA) is a chronic inflammatory, autoimmune polyarthritis, with a prevalence estimated at one percent of the United States(US) population. Serum bilirubin, because of its antioxidant nature, has been conjectured to exert an anti-inflammatory biologic effect. The objective of this study is to discern whether higher serum Total Bilirubin(TBili) levels are protective against RA.

**Methods:**

This is a secondary analysis of National Health and Nutrition Examination Survey (NHANES) data collected between 2003-2006. Study participants completed a comprehensive questionnaire regarding their health history, underwent a physical examination, and had body fluids collected for laboratory studies. In NHANES, to assess for the presence of RA, the following questions were asked: "Doctor ever said you had arthritis?" If so, "Which type of arthritis". Statistical analysis was performed, using SAS version 9.1, proc survey methods. Participant data were adjusted for demographic characteristics as well as risk factors for RA.

**Results:**

NHANES 2003-2006 included 20,470 individuals, chosen as a representative sampling of the entire US population. Exclusion criteria included age less than twenty years or liver dysfunction, defined as history of abnormal liver function tests or liver disease. 8,147 subjects did not have any exclusion criteria and were included in the data analysis. RA is inversely related to the serum level of TBili with an odds ratio of 0.679 (95% CI 0.533-0.865) and remained significant even after adjusting for age, gender, race, education, and tobacco history, with an odds ratio 0.749 (95% CI 0.575 - 0.976).

**Conclusions:**

Our study supports the hypothesis that higher TBili levels are protective against RA. A plausible mechanism for this association would be that the anti-oxidant effects of TBili exert a physiologic anti-inflammatory effect, which provides protection against RA. This explanation is supported by prior studies which show that higher TBili levels are protective against stroke, atherosclerosis, and vasculitis. Further studies are needed to delineate the exact nature of the protective properties of TBili.

**Keywords:**

Bilirubin; Rheumatoid arthritis; Antioxidant; Protective

## Introduction

Despite great advances in drug development for Rheumatoid Arthritis (RA), a large number of patients still tend to rely on alternative treatments such as antioxidant therapy and diet, suggesting that their RA is still not being adequately controlled. New therapeutic approaches targeting the underlying, pathologic inflammation in novel ways have the potential to bridge this therapeutic gap. In delineating the etiologies of inflammation in RA, the generation of reactive oxygen and nitrogen species has been shown to be critical to the genesis of this disease [1, 2]. Thus, modalities that decrease production of reactive, oxidative, inflammatory molecules would have direct disease modifying effects. One approach that has shown potential is vitamin supplementation, such as with Vitamin E [3-5]. However, the literature remains unclear as to whether this theoretical benefit translates into clinical improvement in RA patients.

Total Bilirubin (TBili) has been studied as a chemical with similar, anti-oxidant properties. Unlike with Vitamin E, a growing body of data exists to show that its anti-inflammatory properties do translate into decreased human morbidity [6, 7]. Indeed, literature shows that higher TBili levels correlate with a reduced risk of stroke, atherosclerosis, renal perfusion injuries, and angiotensin-mediated hypertension [8-13]. Despite this robust body of literature showing the benefits of higher TBili levels in varied inflammation-related morbidities, no literature exists investigating how higher TBili levels impact RA.

## Methods

### Survey design

The NHANES survey is a continuous assessment of health and nutrition conducted by the Centers for Disease Control and Prevention (CDC) and its subsidiary, the National Center for Health Statistics (NCHS). Findings from this survey are utilized to determine the prevalence of major diseases and their risk factors. The NHANES survey consists of household interviews, physical and dental evaluations, and body fluid collections, such as blood and urine, of a representative sample of the non-institutionalized, United States population, using a complex, stratified, multistage probability cluster survey design. Older individuals, children, Mexican Americans, and African Americans are purposefully over-represented to insure a pre-specified minimum sample size. Sampling weights are used to adjust the cohort distribution so that it more closely models the US population. Between 2003 and 2006, 20,470 subjects participated in the NHANES survey process. Those individuals less than 20 years old or with liver disease were excluded from data analysis. A total of 8,147 subjects did not meet any exclusion criteria and were included in data analysis.

### Laboratory methods

Serum samples were collected and analyzed in a standardized manner from fasting individuals as described in the White Sands Clinical Laboratory's Collection Procedures and Specimen Requirements Manual 8. Blood drawn from the examinee's arm was processed and stored on dry ice at -70^o^C. The analyses were performed with a Beckman Synchron LX20. The serum TBili concentration was assayed using a timed-endpoint diazo method. This chemical assay is predicated on bilirubin reacting with the diazo reagent, in the presence of caffeine, benzoate, and acetate as accelerators to form azobilirubin. The assay measurement instrument detects changes in light absorbance, at 520 nm wavelength, after the chemical reaction has progressed for a fixed time interval.

### Definition of variables

NHANES survey interviewers completed an extensive two-week training course prior to conducting interviews. A large percentage of the interview is conducted in both English and Spanish. RA was defined based on the subject's response to the following questions: 'Have you ever been told by a doctor that you had arthritis?' and 'What type of arthritis?' Liver disease was defined based on a subject's response to the question 'Have you had liver disease?' Participants who answered 'yes' to the question regarding liver disease or who had AST values greater than 50 ng/ml on serum studies, or ALT values greater than 50 ng/ml on serum studies, were excluded from data analysis. Other co-variables included were self-reported age, gender, race/ethnicity, smoking status, education status, and marital status. BMI was calculated from height and weight measurements, and placed into 3 categories based on the CDC criteria of normal (less than 25), overweight (25 - 29.9) and obese (greater than 30).

### Statistical analysis

NHANES uses a complex, stratified, multi-state probability-cluster sampling survey design. Due to the complexity of this survey's design, we used SAS version 9.1's (Cary, NC) PROCSURVEY methodology. A Jackknife Replacement method was used to estimate the sample variance. Logistic regression models were created to look at the unadjusted association between RA and the TBili. Adjusted regression models, using the variables age, gender, race, marital status, education, smoking and BMI, were also derived.

## Results

**Figure 1.**
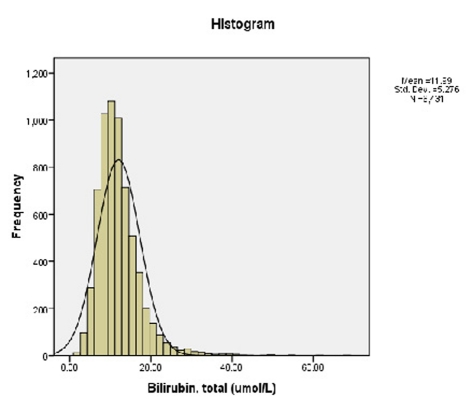
Prevalence of serum TBili among the participants.

Our analysis showed the data to be normally-distributed, with a right tail (Fig. 1). The mean TBili level was 11.99 micromoles per liter with a standard deviation of 5.28.

**Figure 2.**
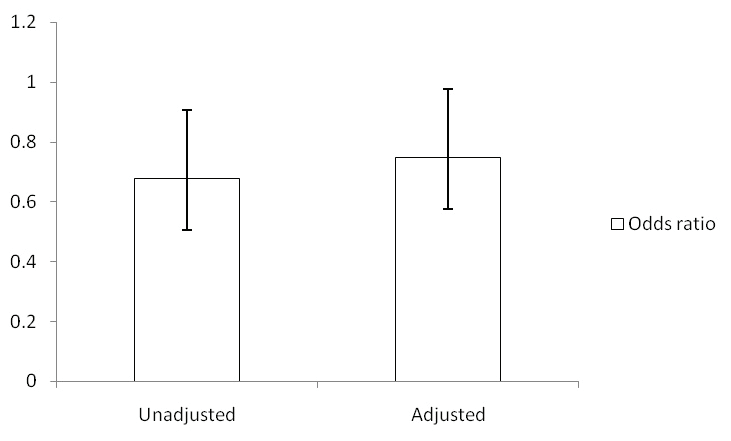
Odds ratio of RA in subjects with higher TBili levels in comparison to subjects with lower TBili levels. Note: odds ratio of RA in lower TBilli subjects defined as 1.

Based on prior studies investigating the disease preventative effects of TBili, our analysis stratified TBili levels as either less than or greater than 11 micromoles per liter [11]. The demographic characteristics of these two groups were then compared (Table 1). With respect to the age-adjusted prevalence, males had higher TBili levels while females tended to have levels less than 11 micromoles per liter. Among participants with a BMI less than 30, a higher proportion of subjects had higher TBili levels.

**Table 1:** Demographic Characteristics (Age Adjusted Prevalence)

	Serum TBili <11micromols/liter	Serum TBili >11micromoles per liter
Age 20-39	46.46% SE 1.14	53.54 SE 1.14
40-59	44.73% SE 1.16	55.27 SE 1.16
60 and above	40.25% SE 1.54	59.74 SE 1.54
Unmarried	49.16% SE 1.24	50.82 SE 1.24
Married	40.85% SE 1.12	59.14 SE 1.12
Edu < high school	52.31% SE 1.90	47.68 SE 1.90
Edu > high school	42.65% SE 0.97	57.34 SE 0.97
Non smoker	43.02% SE 1.24	56.98 SE 1.24
Smoking	45.79% SE 0.95	54.02 SE 0.95
Black	56.78% SE 1.26	43.22 SE 1.26
Non-black	42.73% SE 0.98	57.27 SE 0.98
Male	30.63% SE 1.02	69.37 SE 1.02
Female	56.28% SE 1.27	43.27 SE 1.27
BMI less than 25	43.24% SE 1.45	56.76 SE 1.45
25-29	39.57% SE 1.43	60.43 SE 1.45
30 and above	50.42% SE 1.28	49.58 SE 1.28
No Rheumatoid arthritis	44.07% SE 0.92	55.93 SE 0.92
Rheumatoid arthritis	51.23% SE 3.62	48.77 SE 3.62

With respect to RA, a greater proportion of subjects who did not report any history of RA had a TBili above 11 micromoles per liter (55.93% with SE of 0.92). Although there was a trend toward more patients in the RA stratification also having lower TBili levels (less than 11 micromoles per liter), it did not reach statistical significance. However, a statistically-significant, unadjusted association between higher TBili levels and RA did exist (OR 0.68, 95% CI 0.53 - 0.87). The relationship remained statistically significant after adjusting for age, sex, race, married status, education (Fig. 2), BMI and smoking (OR 0.75, 95% CI 0.58 - 0.98).

## Discussion

Previous studies have suggested that the antioxidant properties of TBili have disease preventative effects with respect to stroke, coronary artery disease and peripheral vascular disease [8-11]. In this study, we found that this inverse relation between higher TBili levels and disease prevalence extends to RA as well.

The exact nature of this protective effect is not clear. We do know that the inflammation inherent to RA results from immunological reactions involving the activation of granulocytes, macrophages and lymphocytes [1]. These inflammatory cells, in the synovial fluid, are in an activated state, releasing oxygen-derived free radicals that are lethal to the joint tissue [2]. One possibility for the protective effect of TBili with regard to RA risk suggests that Bilirubin clears peroxyl radicals, decreases oxidative stress and by binding to serum albumin can prevent in-vitro oxidation of albumin-bound fatty acids [13-15].

Furthermore, unconjugated Bilirubin and Biliverdin have been shown to exhibit immune protective effects on murine liver and cardiac grafts. In the case of Biliverdin, this effect has been shown to be mediated by suppression of IL-2 production, via inhibition of nuclear factor. Recent research has shown that intracellular redox cycling of bilirubin to biliverdin, a metabolite of bilirubin, may be important for cytoprotection [3].

In reviewing the strengths and weaknesses of this study, this study's large sample size would lend itself to increased resolution of small differences in the groups studied. Furthermore, use of data from NHANES facilitates investigation of the interaction among multiple co-variables and its study design facilitates generalization to the US population, as a whole. Since our analysis is based on data derived from a survey, there will be an inherent inability to determine temporal association of higher TBili levels and RA. In addition, this study is limited by the survey participants self-report of RA, possibly leading to inaccurate estimates of disease prevalence based on participant misconceptions regarding their level of health. The laboratory analysis of participant blood did not include fractionation of bilirubin levels. Thus, it is not possible to discern whether the anti-oxidant effects of TBili are a combined phenomenon versus the sole effect of Indirect or Direct Bilirubin.

In conclusion, higher serum TBili levels are negatively associated with RA. These findings introduce the possibility of a therapeutic role of bilirubin as an anti-inflammatory agent, alongside pharmacotherapy, to reduce the process of joint destruction. Further studies, particularly cohort studies, are needed to confirm this association and to investigate approaches to manipulate the TBili level for the patient's benefit.

## References

[1] McInnes IB, Leung BP, Field M, Wei XQ, Huang FP, Sturrock RD, Kinninmonth A (1996). Production of nitric oxide in the synovial membrane of rheumatoid and osteoarthritis patients. J Exp Med.

[2] Remans PH, van Oosterhout M, Smeets TJ, Sanders M, Frederiks WM, Reedquist KA, Tak PP (2005). Intracellular free radical production in synovial T lymphocytes from patients with rheumatoid arthritis. Arthritis Rheum.

[3] Baranano DE, Rao M, Ferris CD, Snyder SH (2002). Biliverdin reductase: a major physiologic cytoprotectant. Proc Natl Acad Sci U S A.

[4] Brambilla D, Mancuso C, Scuderi MR, Bosco P, Cantarella G, Lempereur L, Di Benedetto G (2008). The role of antioxidant supplement in immune system, neoplastic, and neurodegenerative disorders: a point of view for an assessment of the risk/benefit profile. Nutr J.

[5] Edmonds SE, Winyard PG, Guo R, Kidd B, Merry P, Langrish-Smith A, Hansen C (1997). Putative analgesic activity of repeated oral doses of vitamin E in the treatment of rheumatoid arthritis. Results of a prospective placebo controlled double blind trial. Ann Rheum Dis.

[6] Bendich A (1988). Vitamin E and immune functions. Basic Life Sci.

[7] Bosma PJ, Chowdhury JR, Bakker C, Gantla S, de Boer A, Oostra BA, Lindhout D (1995). The genetic basis of the reduced expression of bilirubin UDP-glucuronosyltransferase 1 in Gilbert's syndrome. N Engl J Med.

[8] Adin CA, Croker BP, Agarwal A (2005). Protective effects of exogenous bilirubin on ischemia-reperfusion injury in the isolated, perfused rat kidney. Am J Physiol Renal Physiol.

[9] Endler G, Hamwi A, Sunder-Plassmann R, Exner M, Vukovich T, Mannhalter C, Wojta J (2003). Is low serum bilirubin an independent risk factor for coronary artery disease in men but not in women?. Clin Chem.

[10] Lanone S, Bloc S, Foresti R, Almolki A, Taille C, Callebert J, Conti M (2005). Bilirubin decreases nos2 expression via inhibition of NAD(P)H oxidase: implications for protection against endotoxic shock in rats. FASEB J.

[11] Novotny L, Vitek L (2003). Inverse relationship between serum bilirubin and atherosclerosis in men: a meta-analysis of published studies. Exp Biol Med (Maywood).

[12] Perlstein TS, Pande RL, Creager MA, Weuve J, Beckman JA (2008). Serum total bilirubin level, prevalent stroke, and stroke outcomes: NHANES 1999-2004. Am J Med.

[13] Pflueger A, Croatt AJ, Peterson TE, Smith LA, d'Uscio LV, Katusic ZS, Nath KA (2005). The hyperbilirubinemic Gunn rat is resistant to the pressor effects of angiotensin II. Am J Physiol Renal Physiol.

[14] Sano K, Nakamura H, Matsuo T (1985). Mode of inhibitory action of bilirubin on protein kinase C. Pediatr Res.

[15] Stocker R, Yamamoto Y, McDonagh AF, Glazer AN, Ames BN (1987). Bilirubin is an antioxidant of possible physiological importance. Science.

